# The Same or Different? Convergence of Skin Gambling and Other Gambling Among Children

**DOI:** 10.1007/s10899-019-09840-5

**Published:** 2019-03-09

**Authors:** Heather Wardle

**Affiliations:** London School of Hygiene and Tropical Medicine, 15-17 Tavistock Place, London, WC1H9SH UK

**Keywords:** Gambling, Gaming, Children, Convergence, Survey, Skin betting

## Abstract

There is increasing attention on the introduction of gambling-like practices within video games. Termed convergence, this has been explored from the viewpoint of the product, examining similarities in game/gambling mechanics. Understanding convergence of practice is essential to map the epidemiology of these behaviours, especially among children. This paper focuses on the betting of skins within video games to explore co-occurrence with other forms of gambling among British children aged 11–16. Analysing the British Youth Gambling Survey showed that 39% of children who bet on skins in the past month had also gambled on other activities. Betting on skins and other forms of gambling increased with age and concordance of skin gambling/betting was greatest for those who also gambled online. Among gamblers, those who bet skins had higher rates of at-risk and problem gambling than those who did not (23% vs. 8%), though they had a greater breath of gambling involvement. Skin gambling alone was not significantly associated with at-risk gambling when other forms of gambling activity were taken into account. Skin betting and gambling on other activities cluster together, especially where the medium underpinning the behaviours is the same. Children who engage in both skin gambling/betting and other forms of gambling should be considered at-risk for the experience of harms because of their heightened engagement in gambling and gambling-like activities.

## Introduction

New media and its associated technological infrastructure have created conditions in which forms of gambling can, and increasingly are, being incorporated into digital life and practice (Macey and Hamari 2018a; Griffiths et al. 2013). This is particularly true within video games, which incorporate relatively new and emerging practices that replicate and reproduce gambling-like activities within this media. These practices include loot boxes, where players pay to ‘open’ a virtual box in the hope of it containing in-game items of significantly higher value than their original outlay, or the gambling or betting of ‘skins’ (decorative in-game items) through various mediums (Macey and Hamari 2018a). There has been much consideration of the intersection between video game participation and engagement in other risky practices, including gambling (Macey and Hamari 2018b) and it suggested that video game engagement could serve as a gateway into other gambling activities, though as Macey and Hamari (2018b) point out, evidence on this is mixed.

These studies have tended to focus on the relationship between any form of video game play and gambling behaviour (McBride and Derevensky 2017). However, within video games, an increasing number of gambling-like activities are available, and less consideration has been given to the intersection of these ‘within game’ features with more traditional forms of gambling activity. These features, such as skin gambling/betting or the purchase of loot boxes, are facilitated by micro transactions within video games, whereby players pay to purchase in-game virtual items or access to certain game features. These transactions are an increasingly common and profitable part of the gaming ecosystem (ParentZone 2018; King and Delfabbro 2018). This then facilitates a range of other actions for players, such as the betting or trading of skins, mainly on third party websites. Skins are virtual items earned or purchased within video games, which have their own value within the gaming community. They are decorative items that have no bearing on the outcome of the game but are highly sought after nonetheless and first emerged in 2012 within the game Counter Strike: Global Offensive (ParentZone 2018). Skins are purchased from a digital marketplace and some skin items are more valuable than others, often based on rarity, popularity and potential use (ParentZone 2018; Gambling Commission 2017a). The value of these items, like ‘hard’ currency itself, gold or diamonds (or using seventeenth century examples, tulip bulbs) can fluctuate based on these features. Through third party access to digital marketplaces, where skins are bought and stored, skins can be bet or traded on other websites and thus the virtual value of the skin converted into real currency. These practices are examples of a common phenomenon within digital games, where a range of different actions and industries develop around and extend from the core game (Kerr 2006). With regards to skin betting and gambling, there is ambiguity around the nature of the practice, though the British Gambling Commission (the industry regulator) stated that they consider skins to have real world value and that betting of them represents a ‘money’s worth prize’ (Gambling Commission 2017a). Skins therefore function as a form of crypto-currency with their own value but can also be converted into ‘real world’ currency. This suggests that the betting of these items extends beyond game play and could be considered gambling conducted via processes and websites where there is no robust age verification in place and which are complex to regulate.

Concern about skin betting practices have been heightened with respect to children and the potential role they may play in shaping problematic gaming and/or gambling practices (Gainsbury et al. 2015; King and Delfabbro 2018). Described as convergence between gaming and gambling, three interlocking challenges have been considered: that these ‘convergent’ practices could prompt children to gamble more generally as a form of gateway activity, that engagement in this form of activity alone could be harmful and that these practices normalise gambling for a cohort of children (ParentZone 2018).

In Britain, as elsewhere, children are singled out for specific regulatory protections from gambling with legal age limits placed on most commercial forms. Nonetheless, it is estimated that 12% of children aged 11–15 have participated in some form of gambling activity in the past week, with over half of this activity being on commercial and (technically) legally restricted forms (Wardle 2018a). Furthermore, it is estimated that around 0.8% of children aged 11–15 in Great Britain experience problems with their gambling behaviour and early onset of gambling in childhood is a known risk factor for subsequent problems (Blinn-Pike et al. 2010; Forrest and McHale 2018). It is in this context that the challenges of these seemingly convergent digital practices are raised as they are viewed as providing the means for children to gamble and access gambling content. Politicians are giving this increasing attention, with questions asked by UK parliamentarians about the impact of skin betting on underage children (UK Parliament 2018).

The betting and gambling of skins is popular among children and young people. A recent survey of 13–18 years olds in Great Britain estimated that 10% had ever gambled or bet skins (ParentZone 2018). In 2017, report by the British Gambling Commission found that 11% of 11–16 years olds had ever bet skins, and that 4% had done so in the past week. This made skin gambling/betting as popular as playing on fruit/slot machines and more popular than most other ‘traditional’ forms of gambling activity (Gambling Commission 2017b). Yet to date, there has been (to the author’s knowledge) little empirical examination of the extent to which skin gambling among children is combined with other forms of gambling; empirical insight which is needed to explore the whether gambling and gaming are mutually reinforcing consumptive practices, and if so, to what extent.

## Objectives and Hypotheses

Understanding the potential impact of engagement in skin gambling among children requires a greater consideration of children’s behaviours in order to map the basic epidemiology of practices. To date, notions of convergence between gambling-like activities and more traditional forms of gambling have tended to be examined by focusing on the products, with researchers noting the similarities of these practices, their common structural features and reward system mechanisms (King and Delfabbro 2018; McBride and Derevensky 2017). It is, however, vitally important to understand convergence of behaviours, especially if theories about one practice leading to another are to be better explored. This research uses nationally representative data of children aged 11–16 to explore this and to estimate:

- the extent to which skin gambling and betting among 11–16 years olds is combined with other, more ‘traditional’ forms of gambling;
- how the prevalence of skin gambling (alone and in combination with other forms of gambling) varies by different socio-demographic and economic characteristics; and
- whether rates of problem and at-risk gambling vary by engagement in skin betting/gambling.

It is hypothesised that skin gambling and other forms ‘traditional’ forms of gambling will cluster together, given the similarities between the practices meaning that those who are interested in one form of practice are also likely to be interested in others (H1). It is also hypothesised that this clustering will be socially patterned, being more common among certain types of children, especially boys (H2) and those from more disadvantage backgrounds (H3). Finally, it is hypothesised that children who participate in skin gambling and other forms of gambling will display greater levels of at-risk or problem gambling (H4), as a function of their greater involvement with gambling and gambling-like activities more generally.

## Methods

### Data

Secondary analysis of the 2017 Youth Gambling Survey, conducted for the British Gambling Commission by Ipsos Mori via their youth omnibus survey, was undertaken. The youth omnibus collects survey information from a random sample of school-aged children in years 7–11 on a range of topics (funded by different clients). The Gambling Commission funds a subsection of the questionnaire to collect some data about gambling behaviour. Overall, 446 secondary schools were randomly chosen from the Edubase list in England and Wales and from a listing provided by the Scottish Government in Scotland. The school sample was stratified by Government Office Region and, within each stratum, further stratified by Local Authority, area deprivation and school size. Within each participating school, one curriculum year group (Year 7–Year 11) was selected to participate at random for each school. All members of the randomly-selected class group were asked to fill out a paper self-completion survey. Overall, 103 selected schools participated, giving a school-based response rate of 23%. Questionnaires were obtained from 2881 pupils aged 11–16 (Ipsos 2017).

### Measures

#### Skin Betting Measures

In 2017, four questions about video games and skin betting and gambling were included for the first time. The following questions were asked: whether children ever played computer games or game-apps these days; those who had were then asked if they were aware of betting with in-game items and whether they had personally done so. Those who had bet or gambled using skins were asked how often they had done so (within the past 7 days, month or past year). Questions asked about skin betting were preceded by this introduction: ‘when playing computer games/apps it is sometimes possible to collect in-game items (e.g. weapons, power-ups and tokens). For some games, it is possible to bet these in-game items for the chance to win more of them.’ This is the definition of skin gambling/betting used within the survey and thus is the definition for the analysis presented in this paper. Using this information, children who had bet using skins in the past month were identified.

#### Gambling Measures

All children were asked whether they had used their *own* money in the past week on one of 14 forms of gambling activity, ranging from purchasing lottery tickets, scratchcards or private betting to betting in bookmakers, casinos or online gambling or betting. All children were also asked how often in the past year they had spent their own money on each of the following: lottery tickets, scratchcards, fruit machines, bingo, online gambling or betting and private betting or gambling with friends. For this analysis, those who had gambled on at least one of these six activities on a monthly basis and anyone who had gambled in the past week were defined as ‘past month gamblers’. The absence of more detailed frequency data for some forms of gambling (for example betting in bookmakers) may mean there are some false positives within the non-past month gambler group, though the forms of gambling excluded were very low prevalence (Gambling Commission 2017b). Gambling problems were measured using the DSM-IV-J-MR instrument. This was developed and validated by Sue Fisher specifically to assess gambling problems among adolescences (Fisher 2000). Responses to 12 items are scored and summed out of a maximum of 10 (there are three items where a score of one is given if anyone of the three behaviours is endorsed). A score of 4 or more indicates problem gambling and a score of 2–3 indicates at-risk gambling (Fisher 2000; Olason et al. 2006; Castrén et al. 2015). Because of small base sizes (problem gambling n = 25), the at-risk and problem gambling categories have been combined in this analysis and, following Castrén et al. (2015) termed at-risk or problem gambling (Castrén et al. 2015).

#### Skin Betting and Gambling Measures

Using the measures described above, all children were allocated to one of the following groups: had bet with skins and gambled on other activities in the past month; had bet with skins in the past month only; had gambled on other activities in the past month only, had participated in neither in the past month. This was undertaken for participation in all gambling activities combined and for each of the six individual gambling activities where frequency data was available. These variables were used to explore the extent to which skin gambling may co-occur with certain types of gambling activity as well as gambling overall.

#### Socio-demographic/Economic Measures

The youth omnibus survey collects very limited details of children’s socio-economic or demographic circumstances. This is partly because it is a school-based survey and limited questions can be asked about the home circumstances of their parents and families. It is also partly because it is an omnibus study and questionnaire space is reserved for paying clients. This is common among most surveys of children conducted within this setting. Therefore, demographic and socio-economic measures are limited but do include some key measures known to be associated with children’s gambling behaviour, namely age, sex, ethnicity, self-rated academic performance and a measure of low-income status, represented by receipt of free school meals (Blinn-Pike et al. 2010; Forrest and McHale 2018). Because of small base sizes, age was grouped into 2-year bands and ethnicity grouped into White/White British; Asian/Asian British, Black/Black British and mixed/other. Children reported how well they felt they were doing at school on a four point scale and responses grouped into those doing well versus not doing well. Children were asked whether they were in receipt of free school meals. Free school meals are only available to parents in receipt of income-based benefits and thus act as a proxy for identifying low-income families.

### Analyses

Bi-variate associations between the prevalence of skin gambling (alone and in combination with other forms of gambling) and socio-demographic/economic characteristics were produced using SPSS’s complex survey module. For bi-variate analyses, the complex survey function produces an adjusted Wald’s F-test as its default test of significance, which assesses the extent to which the independent variable (prevalence of skin betting, for example) varies by the dependent variables (age or gender, for example), whilst taking into account the survey weighting, stratification and clustering of children within classes (Rao and Scott 1984). All *p* values cited in the tables relate to this type of statistical testing. Following Graham et al. (2014), observed-expected ratios were computed to assess the extent to which skin gambling and other forms of gambling cluster together. Observed-expected ratios are interpreted relative to their confidence intervals. An observed-expected ratio greater than one, with a confidence interval that does not straddle 1, represents a higher prevalence than would be expected if the behaviours were independent and indicates clustering of behaviours. Finally, two multivariate logistic regressions were run to (a) examine whether certain forms of gambling were associated with skin gambling in the past month and (b) whether skin gambling was associated with at-risk gambling, once other forms of gambling engagement was taken into account. Checks for collinearity between individual forms of gambling activities were undertaken [assessment of phi correlations for binary data and variance inflation factor (VIF) diagnostic tests] and found to be minimal (available on request from the author). Both models also controlled for age, sex and academic attainment as bi-variate analyses showed these were associated with skin gambling. Regression models were produced using Stata v15, and took into account the survey weights and complex study design. Missing data was minimal and excluded from analyses. Ethical approval was provided by the London School of Hygiene and Tropical Medicine Ethics’ committee (Ref. 15960).

## Results

### Engagement in Individual Activities

Table 1 shows overall prevalence of participating in skin gambling and other forms of gambling in the past month. Overall, 7% (95% CI 5.5, 7.5) of children had bet with skins and 16% (95% CI 13.6, 16.4) had gambled on other forms of gambling activity in the past month. Prevalence of past month betting for other gambling activities ranged from 2% (95% CI 1.7, 2.7) for online gambling or playing bingo to 8% (95% CI 7.3, 9.3) for betting with friends. Skin betting was the second most popular form of activity among children overall and among boys, it was the most prevalent activity of those reported. Among girls, it was one of the least popular activities undertaken.

**Table 1:** Participation in different forms of activity in the past month, by sex

	Boys (%)	Girls (%)	All (%)
Bet with skins*	12	1	7
Bought National Lottery tickets**	3	2	3
Bought scratchcards	5	4	5
Played fruit/slot machines*	7	4	5
Played bingo for money	2	1	2
Gambled online for money (includes National Lottery Instant Win games)**	4	1	2
Bet with friends for money*	11	5	8
Any form of gambling (excluding skin betting)**	21	10	16
Bases: unweighted	1312	1412	2760

*p < 0.01; **p < 0.05

Rates of skin betting rates rose with age, rising from 4% (95% CI 2.3, 4.9) for those aged 11–12 to 7% (95% CI 5.4, 9.4) for those aged 15–16. Notably, rates of gambling on other activities did not vary significantly by age. Skin gambling did not vary significantly by ethnicity, self-reported academic performance or receipt of free school meals (Table 2).

**Table 2:** Combination of skin betting and other gambling by socio-demographic/economic characteristics

	Gambled on skins and other forms	Skin betting only	Other gambling only	Neither	Any skin gambling in past month	Any gambling in past month	Bases: unweighted
Sex^a^							
Boys (%)	4	7	16	72	12	21	1198
Girls (%)	1	1	9	89	1	10	1333
All (%)	3	4	13	81	7	16	2563
Age^b^							
11–12 (%)	1	3	13	83	4	14	796
13–14 (%)	3	5	14	78	8	17	1101
15–16 (%)	3	4	11	81	7	14	666
Ethnic group							
White/White British (%)	3	4	13	81	7	15	1985
Asian/Asian British (%)	2	3	11	84	5	13	110
Black/Black British (%)	2	2	16	80	4	18	275
Mixed/other (%)	3	6	10	80	10	13	160
Academic performance^c^							
Doing well (%)	2	4	12	82	6	14	2078
Not doing well (%)	6	3	16	74	10	22	303
In receipt of free school meals							
Yes (%)	3	3	12	83	6	14	241
No (%)	3	4	12	81	7	15	2178

^a^For prevalence of betting on both skins and other forms of gambling; betting on skins and betting on other forms of gambling significantly different *p* < 0.05

^b^For prevalence of any skin betting in past month or betting on skins *p* < 0.05

^c^For prevalence of betting on both skins and gambling on other things or any gambling *p* < 0.05

### Concordance of Skin Betting and Gambling on Other Activities

Overall, 3% (95% CI 1.9, 3.1) of children reported gambling on both skins and other forms of gambling activity in the past month. The observed-expected (O/E) ratio for both skin gambling and gambling on other activities among all children was 2.5 (95% CI 1.9, 3.2, see Table 3). Among skin bettors, 39% (95% CI 31.6, 46.4) had also bet on some other form of gambling whilst 61% had bet on skins alone. To look at this another way, 16% (95% CI 12.4, 19.6) of children who had gambled on other forms of activity had also bet on skins in the past month.

**Table 3:** Observed/expected (O/E) ratios for combinations of skin betting and individual gambling activities

	Did neither activity	Skin betting only	Other individual gambling activity only	Bet with skins and gambled on other individual activity	Bases: unweighted
O/E ratios for skin betting and buying National Lottery tickets	1.0 (1.0, 1.0)	1.0 (0.8, 1.1)	0.9 (0.7, 1.1)	2.7 (1.1, 4.2)	2490
O/E ratios for skin betting and buying scratchcards	1.0 (1.0, 1.0)	0.9 (0.8, 1.1)	0.9 (0.7, 1.1)	2.8 (1.6, 4.0)	2489
O/E ratios for skin betting and playing fruit/slot machines	1.0 (1.0, 1.1)	0.9 (0.7, 1.0)	0.8 (0.7, 1.0)	3.3 (2.1, 4.5)	2505
O/E ratios for skin betting and playing bingo for money	1.0 (1.0, 1.1)	1.0 (0.8, 1.1)	0.9 (0.6, 1.1)	3.2 (1.3, 5.1)	2485
O/E ratios for skin betting and gambling online for money	1.0 (1.0, 1.0)	0.9 (0.7, 1.0)	0.7 (0.4, 0.9)	5.9 (3.3, 8.5)	2481
O/E ratios for skin betting and betting with friends for money	1.0 (1.0, 1.1)	0.8 (0.7, 1.0)	0.9 (0.7, 1.0)	2.9 (2.0, 3.8)	2481
O/E ratios for skin betting and any form of gambling in the past month	1.0 (1.0, 1.1)	0.7 (0.6, 0.9)	0.9 (0.8, 1.0)	2.5 (1.9, 3.1)	2563

Observed-expected ratios between skin betting/gambling and gambling individually on each of the six main activities all indicated a significant level of clustering than would be expected given their population prevalence. The observed-expected ratios for skin betting/gambling and gambling online were almost six times higher than expected (O/E = 5.9, 95% CI 3.3, 8.5), whilst for fruit/slot machine betting it was over three times higher than expected (O/E = 3.3, 95% CI 2.1, 4.5). Among those who gambled online in the past month, 37% had also gambled with or bet skins.

The strength of the association between online gambling, fruit/slot machine gambling and skin betting was confirmed in multivariate regression analysis. The odds of having gambled or bet skins in the past month were 3.8 (95% CI 1.1–12.8) times higher among those who had also gambled online than those who had not, even after engagement in other forms of gambling, age, sex and academic attainment were taken into account. Odds of skin gambling were also higher among those who had bet on fruit/slot machines in the past year (2.7; 95% CI 1.4–5.2). However, all other individual forms of gambling activity were not associated with past month skin gambling in the regression model (Table 4).

**Table 4:** Odds of betting or gambling on skins in the past month

	Odds ratio	Confidence interval: lower	Confidence interval: upper
Sex (*p* < 0.05)			
Boys	1		
Girls	0.1	0.1	0.2
Age (*p* < 0.01)			
11–12	1		
13–14	2.1	1.3	3.5
15–16	2.1	1.2	3.7
Academic attainment (*p* = 0.39)			
Not doing very well	1		
Doing well	1.5	0.8	2.8
Gambling activities in last month^a^			
Bet on lotteries (*p* = 0.38)	0.5	0.1	2.4
Bet on scratchcards (*p* = 0.36)	1.5	0.6	3.8
Bet on fruit/slot machines (*p* < 0.01)	2.7	1.4	5.2
Bet on bingo (*p* = 0.74)	0.8	0.3	2.4
Bet on online (*p* < 0.05)	3.8	1.1	12.8
Bet privately/played games for money (*p* < 0.07)	1.7	1.0	2.9

^a^Each gambling activity was entered into the model individually and odds presented are relative to those who did not do each activity

### Patterns of Convergence by Socio-demographic/Economic Characteristics

Boys were more likely than girls to report gambling on both skins and other forms of gambling, though this is unsurprising given the increased preference for both individual activities among boys. However, observed-expected ratios were higher for girls suggesting that despite these being very low prevalence activities for girls overall, they were highly likely to cluster together. The concordance of gambling both on skins and other activities increased with age, being higher among those aged 13–16 than those aged 11–12. Observed-expected ratios for both skin betting and gambling on other activities rose from 1.6 among those aged 11–12 [though the 95% CI straddled 1 (0.4–2.8)] to 2.4 for those aged 15–16 (95% CI 1.7–4.3). Gambling on both skins and other activities in the past month was higher among those who reported that they were not doing well at school than those who were doing well, though observed/expected ratios suggested that skin gambling and other forms of gambling clustered for both groups [O/E for those doing well = 2.5 (95% CI 1.6–3.0); not doing well = 2.9 (95% CI 1.7–4.2)] (Table 5).

**Table 5:** Observed/expected ratios for combinations of skin betting and gambling behaviour by socio-demographic/economic characteristics

	Neither bet with skins or gambled	Skin betting only	Other gambling only	Bet with skins and gambled on other activities	Bases: unweighted
Sex					
Boys	1.0 (1.0, 1.1)	0.8 (0.6, 0.9)	0.9 (0.8, 1.0)	1.8 (1.3, 2.3)	1198
Girls	1 (0.9, 1.1)	0.7 (0.3, 1.0)	1 (0.8, 1.1)	4.2 (1.3, 7.2)	1333
Age					
11–12	1.0 (0.9, 1.1)	0.9 (0.5, 1.3)	1 (0.8, 1.2)	1.6 (0.4, 2.8)	796
13–14	1.0 (0.9, 1.1)	0.7 (0.4, 0.9)	0.8 (0.6, 1.0)	3 (1.7, 4.3)	1101
15–16	1.0 (0.9, 1.1)	0.7 (0.4, 0.9)	0.9 (0.6, 1.0)	2.4 (1.7, 4.3)	666
Ethnic group					
White/White British	1.0 (1.0, 1.1)	0.7 (0.6, 0.9)	0.9 (0.8, 1.0)	2.5 (1.9, 3.2)	1985
Black/Black British	*	*	*	*	110
Asian/Asian British	1.0 (0.9, 1.1)	0.7 (0.2, 1.0)	0.9 (0.7, 1.2)	2.5 (0.3, 4.7)	275
Mixed/other	*	*	*	*	160
Academic performance					
Doing well	1.0 (1.0, 1.1)	0.8 (0.6, 0.9)	0.9 (0.8, 1.0)	2.3 (1.6, 3.0)	2078
Not doing well	1.1 (0.9, 1.2)	0.4 (0.2, 0.7)	0.8 (0.6, 1.0)	2.9 (1.7, 4.2)	303
Receipt of free school meals					
Yes	1.0 (0.9, 1.2)	0.6 (0.2, 1.1)	0.9 (0.5, 1.2)	3.2 (0.7, 5.7)	241
No	1.0 (1.0, 1.1)	0.7 (0.6, 0.9)	0.9 (0.8, 1.0)	2.5 (1.9, 3.2)	2178

*Estimates not shown because of small cell sizes

^Because of rounding, the lower CI in some cells looks the same at the observed-expected ratio

### At-Risk/Problem Gambling and Gambling Involvement Among Gamblers

Those who gambled/bet on skins and other types of gambling participated in a greater number of gambling activities (excluding skin gambling/betting), on average, than those who only gambled on other things. At-risk and problem gambling rates were significantly higher among those who had both bet with skins and engaged in other forms of gambling activity in the past month (23%, 95% CI 12.7–34.3) than those who had gambled on other activities alone (8%, 95% CI 4.7–10.5). However, in the multivariate logistic regression model, skin gambling or betting was not associated with at-risk gambling once engagement in other individual gambling activities was taken into account (Tables 6, 7).

**Table 6:** Gambling behaviour characteristics among different types of gambler

	Bet with skins and gambled on other activities (%)	Other gambling only (%)
At risk and problem gambling according to the DSM-IV-MR-J		
Non gambler/non-at risk or problem gambler (DSM-IV-MR-J score of 0/1)**	77	92
At risk or problem gambler (DSM-IV-MR-J score of 2 +)**	23	8
Number of other gambling activities undertaken (excluding skin gambling/betting)		
Mean number of gambling activities undertaken*	2.5	1.9
Median*	2.0	1.0
Bases: unweighted	59	324

*p < 0.05; **p < 0.01

**Table 7:** Odds of being an at-risk gambler (DSM-IV-MJ score 2 +)

	Odds ratio	Confidence interval: lower	Confidence interval: upper
Sex (*p* = 0.07)			
Boys	1		
Girls	0.3	0.1	1.1
Age (*p* = 0.26)			
11–12	1		
13–14	2.0	0.4	10.4
15–16	2.8	0.7	11.3
Academic attainment (*p* = 0.09)			
Not doing very well	1		
Doing well	1.5	0.8	2.8
Skin gambling in past month (*p* = 0.16)			
No	1		
Yes	2.2	0.7	6.3
Gambling activities in last month^a^			
Bet on lotteries (*p* = 0.48)	2.4	0.2	29.1
Bet on scratchcards (*p* = 0.29)	2.4	0.5	11.8
Bet on fruit/slot machines (*p* = 0.14)	2.6	0.7	9.4
Bet on bingo (*p* = 0.38)	2.1	0.4	11.3
Bet on online (*p* < 0.01)	8.4	1.9	37.4
Bet privately/played games for money (*p* < 0.05)	3.7	1.0	13.7

^a^Each gambling activity was entered into the model individually and odds presented are relative to those who did not do each activity

## Discussion

Both skin gambling/betting and gambling on other activities were relatively common among British children aged 11–16, despite some legal restrictions on participation. In Britain, participation on most forms of commercial gambling, including the National Lottery, is age prohibited yet many children still find ways to access these activities, with over half of children’s gambling activity estimated to be on age-restricted forms (Wardle 2018a, b). Playing video games is even more common among this age group and among boys, the gambling or betting of skins was the most prevalent form of ‘gambling’ activity. Evidence from this analysis shows that there is some overlap in who gambles or bets with skins and who takes part in other forms of gambling (confirming hypothesis 1), with 3% of children aged 11–16 saying that they did both. Whilst this may seem like a small number, this equates to around 100,000 children aged 11–16 in Britain. Furthermore, observed/expected ratios show that these two behaviours co-occur more than would be expected given their independent population prevalence, indicating greater overlap between these behaviours than is expected. Notably, the greatest level of overlap was between skin betting and gambling and other forms of online betting or gambling. This is perhaps unsurprising, given the common media underpinning these consumptions. This therefore supports the notion of a ‘convergence’ in behaviours among some children who are engaging in both activities. These patterns of behaviour ‘convergence’ were greatest for boys, older children and those who felt they were doing less well at school, confirming hypothesis 2. However, there was little evidence that this clustering of behaviour occurred disproportionately among those from more disadvantage backgrounds. This may be related to the measure (receipt of free school meals) used to proxy low income households.

However, the evidence is not unequivocal. The most common pattern among those who bet or gambled with skins was that they did not also engage in other forms of gambling. At younger age groups, children tended either to bet on skins or to gamble on other things, if they did this at all. Among older children, skin gambling/betting was more likely to be combined with gambling on other activities, though half of skin gamblers did this activity alone. This suggests a need for greater clarity when talking about processes of convergence between gambling and gaming. As Macey and Hamari (2018a) have highlighted, there is often a tendency with newly emerging consumptive practices to view them in silos rather than to situate them within the broader context of existing behaviours. This paper attempts to address this issue and suggests that there are four distinct groups of children: the majority who engage in neither skin gambling or other forms of gambling; a significant minority who gamble but do not bet with skins (which includes a disproportionate number of girls given their lesser propensity to play video games); a minority who only bet or gambled skins and a further minority who bet and gambled skins and gambled on other things. For the vast majority of children, these behaviours are not converging simply because do not engage in these practices; yet for a minority they are and these behaviours cluster.

Notably, rates of at-risk and problem gambling were highest among gamblers who also engaged in skin gambling/betting (confirming hypothesis 4). This is to be expected. By definition, those engaging in both skin gambling/betting and other forms of gambling have higher levels of gambling involvement because they both gambled on traditional forms of gambling and engaged in a similar practice within video games. However, it is also evident that this group were also more involved in ‘traditional’ forms of gambling alone, with the average number of traditional forms of gambling undertaken being higher among this group also. Involvement theory postulates that the more someone engages in gambling the more likely it is that that they will experience harm from that engagement. This is often explored using the number of gambling activities someone undertakes as a measure of their breadth of gambling engagement (LaPlante et al. 2014; Dixon et al. 2016). The results of the regression analysis showed that the relationship between skin gambling and at-risk gambling attenuated once involvement in a number of other forms of gambling was taken into account. This suggests that it is the combination of skin gambling with other forms that needs further consideration. Therefore, whilst children who gamble with skins as well as other forms of gambling should be considered a high-risk group for the attendant experience of harms, this is likely related to their broader gambling repertoires than their engagement in skin/gambling or betting alone.

Notions of convergence underpin much academic thought about the seemingly mutually reinforcing practices of gaming and gambling. This has tended to approach this issue through analysis of the product, with examples of gambling-like practices embedded within the video game eco-system heralded as examples of convergent of practice and activities. However, there is notable conceptual ambiguity around the demarcation of gambling and play (Caillios 1958; Juul 2003), with some theorists querying where gaming stops and gambling begins. It is therefore important to assess the extent to which these are shared consumptive practices among individuals. This is especially so with children who have been subject to much concern around these developments. Whilst this paper provides some evidence that these behaviour co-occur for some, it does not explore how and why this occurs or, indeed, what type of practice children believe skin gambling to be. Previous research on young people’s perceptions of gambling and gaming noted considerable ambiguity around how young people understand and define gambling activities (Korn 2005; Skinner et al. 2004). This ambiguity may arguably be heightened among children specifically because of the different values they attach to objects in lieu of access to monetary resources (Wardle 2018b). It is imperative, therefore, to understand how children themselves differentiate these consumptive practices and the meanings they attach to them.

## Limitations

Analyses presented are based on self-reported behaviours from a survey of school-aged children and inherits the attendant issues of this methodology. It is secondary analysis, meaning that the analysis presented is limited to the questions designed and funded by the original survey commissioners (for example, only four questions being asked about video games and skin gambling/betting). Only a very limited number of socio-economic characteristics were included in the original survey, limiting the extent to which it is possible to explore how behaviours vary among different types of children. This also limits the range of covariates available to include in the multivariate models and caution should be taken not to view these as models exploring the full range of factors associated with either skin gambling or at-risk gambling. They are presented to give greater descriptive insight into the relationships highlighted through the bi-variate analyses. The definition of skin gambling/betting used is broad and is likely to include private betting/gambling among peers as well as the betting and gambling of skins on third party websites. However, as the definition of gambling used in the survey also includes betting and gambling for money among peers, these are comparable. There is no data about the sequence of activities, only that they were undertaken at broadly the same time. The measure used to represent past month gambling is likely to slightly under-estimate gambling behaviour as frequency of gambling was only collected for six main forms of gambling and excluded less prevalent forms (for example, betting in a bookmakers). Finally, the digital world is fast moving and new products and practices emerge within a short pace of time. Whilst this data was collected in 2017, meaning it is relatively recent, it is possible that the digital landscape has changed in the intervening period.

## Conclusion

Convergence of digital practices is often examined via the lens of the product, where consideration is given to how seemingly similar practices are transferred from one medium to another. Whilst theories about the demarcation between gambling and gaming may be contested, the assimilation of gambling cues within gaming practices and ambiguity about where gaming ends and gambling begins cannot be denied. When considering these issues, it is vital to understand how such conceptual ambiguity manifests in everyday consumption and practice. This paper has shown that, among children, whilst gambling and gaming behaviours do cluster, and do so more for some groups than others, there is also a sizeable majority of children who engage in neither activity or who do one but not the other. This paper also provides some evidence of co-occurring practices among children, especially those conducted through the same medium, where there is a high level of concordance between skin gambling/betting and online gambling. Children who engage in both skin gambling/betting and other forms of gambling should be considered an at-risk group for the experience of harms because of their heightened engagement in gambling and gambling-like activities.
